# Carbonyl reductase as a significant predictor of survival and lymph node metastasis in epithelial ovarian cancer

**DOI:** 10.1054/bjoc.2001.2034

**Published:** 2001-09

**Authors:** M Umemoto, Y Yokoyama, S Sato, S Tsuchida, F Al-Mulla, Y Saito

**Affiliations:** Department of Obstetrics and Gynecology, Hirosaki University School of Medicine, 5-Zaifu-cho, Hirosaki, 036-8562, Japan; Second Department of Biochemistry, Hirosaki University School of Medicine, 5-Zaifu-cho, Hirosaki, 036-8562, Japan; Department of Pathology, University of Kuwait, PO Box 24923, Safat, 13110, Kuwait

**Keywords:** carbonyl reductase, epithelial ovarian cancer, prognosis, lymph node metastasis

## Abstract

We have recently reported a novel function for carbonyl reductase (CR), namely, its ability to modulate the metastatic potential of malignant mouse cells. Because there are currently no data addressing a similar function for CR in human cancers, the aim of this study was to assess a correlation between survival and metastasis, and CR level in epithelial ovarian cancer. Using anti-CR antibody, immunohistochemical staining was performed on 73 epithelial ovarian cancers, 13 borderline malignant tumours, and 25 benign ovarian tumours for a total of 111 specimens. The combined rate for strongly and weakly positive reactions for CR was 32.0% for benign tumours, 38.5% for borderline malignant tumours, and 61.6% for ovarian cancers. The CR-positive rate was 35.7% (weakly positive alone) for ovarian cancers with retroperitoneal lymph node (RLN) metastasis and 67.8% for those without RLN metastasis (*P*< 0.05). The 5-year survival rate was 62.7% for the patients with CR-negative cancer and 86.1% for those with CR-positive cancer (*P*< 0.05). The present results indicate that decreased CR expression in epithelial ovarian cancer is associated with RLN metastasis and poor survival.© 2001 Cancer Research Campaignhttp://www.bjcancer.com


British Journal of Cancer (2001) 85(7), 1032–1036
© 2001 Cancer Research Campaign
doi: 10.1054/ bjoc.2001.2034, available online at http://www.idealibrary.com on IDEAL
http://www.bjcancer.com

### Carbonyl reductase as a significant predictor of survival and lymph node metastasis in epithelial ovarian cancer

**M Umemoto[1], Y Yokoyama[1], S Sato[1], S Tsuchida[2], F Al-Mulla[3] and Y Saito[1]**

[1]Department of Obstetrics and Gynecology, Hirosaki University School of Medicine, 5-Zaifu-cho, Hirosaki, 036-8562, Japan; [2]Second Department of Biochemistry, Hirosaki University School of Medicine, 5-Zaifu-cho, Hirosaki, 036-8562, Japan; [3]Department of Pathology, University of Kuwait, PO Box 24923, Safat, 13110, Kuwait

**Summary** We have recently reported a novel function for carbonyl reductase (CR), namely, its ability to modulate the metastatic potential of malignant mouse cells. Because there are currently no data addressing a similar function for CR in human cancers, the aim of this study was to assess a correlation between survival and metastasis, and CR level in epithelial ovarian cancer. Using anti-CR antibody, immunohistochemical staining was performed on 73 epithelial ovarian cancers, 13 borderline malignant tumours, and 25 benign ovarian tumours for a total of 111 specimens. The combined rate for strongly and weakly positive reactions for CR was 32.0% for benign tumours, 38.5% for borderline malignant tumours, and 61.6% for ovarian cancers. The CR-positive rate was 35.7% (weakly positive alone) for ovarian cancers with retroperitoneal lymph node (RLN) metastasis and 67.8% for those without RLN metastasis ($P < 0.05$). The 5-year survival rate was 62.7% for the patients with CR-negative cancer and 86.1% for those with CR-positive cancer ($P < 0.05$). The present results indicate that decreased CR expression in epithelial ovarian cancer is associated with RLN metastasis and poor survival. © 2001 Cancer Research Campaign http://www.bjcancer.com

**Keyword**: carbonyl reductase; epithelial ovarian cancer; prognosis; lymph node metastasis

Survival rates for patients with epithelial ovarian cancer have shown some improvement in the past decade but remain unsatisfactory (Yokoyama et al, 1999). Generally accepted clinical and pathological prognostic parameters for epithelial ovarian cancers are stage, histologic subtypes and grades, and residual tumour after cytoreductive surgery (Yokoyama et al, 1999). Although biological and genetic parameters, such as macrophage colony-stimulating factor (Chambers et al, 1997), vascular endothelial growth factor (VEGF) (Tempfer et al, 1998), p53 (Werness et al, 1999), and other oncogenes (Silverberg, 1999) have recently been identified as prognostic factors in epithelial ovarian cancers, there is a lack of clinically useful molecular markers for assessing prognosis in ovarian cancer.

Loss of carbonyl reductase (CR) expression in a mouse lung adenocarcinoma cell line has been demonstrated to augment the cells' metastatic capacity (Ismail et al, 2000). Moreover, recent immunohistochemical study has revealed that the combined rate for weakly positive and negative reactions for CR was proportional to the degree of dedifferentiation in hepatocellular cancers (Suto et al, 1999). These results suggest the possibility that decrease or loss of CR by some mechanism might render the tumour cells more malignant than their intrinsic nature.

CR is a cytosolic monomeric, NADPH-dependent oxidoreductase with broad specificity for carbonyl compounds (Lopez de Cerain et al, 1999). It has been isolated from a number of human tissues: brain, liver, breast, ovary, and placenta (Wermuth, 1981; Wermuth et al, 1988; Iwata et al, 1990), and has been therefore implicated in the metabolism of a variety of endogenous and xenobiotic carbonyl compounds: prostaglandins (Lee and Levine, 1974) and anthracycline antibiotics such as daunorubicin (Ahmed et al, 1978). Prostaglandins have been demonstrated not only to modulate apoptosis and bcl-2 expression (Sheng et al, 1998) but also to induce angiogenesis (Tsujii et al, 1998).

In the present study, the expression of CR in epithelial ovarian cancer tissues was immunohistochemically investigated in order to examine the relationship between its expression and clinical-pathological prognostic factors and chemosensitivity.

*Received 8 February 2001*
*Revised 31 May 2001*
*Accepted 12 June 2001*

*Correspondence to:* Y Yokoyama

### MATERIALS AND METHODS

### Patients and tissues

Fresh surgical specimens of ovarian cancer were obtained from 73 patients who were treated at Hirosaki University Hospital between April of 1988 and December of 1998, after informed consent had been obtained. All patients were surgically staged in accordance with the 1988 International Federation of Gynecology and Obstetrics (FIGO) criteria. Namely, they underwent surgery for a total hysterectomy, bilateral salpingo-oophorectomy, partial omentectomy, appendectomy, and pelvic and para-aortic lymphadenectomies. The breakdown for stages of ovarian cancer consisted of 36 patients with stage I, 9 with stage II, 21 with stage III, 7 with stage IV. Histological types were classified into 30 cases with serous cystadenocarcinoma, 17 with mucinous cystadenocarcinoma, 15 with endometrioid adenocarcinoma, 8 with clear cell adenocarcinoma, 3 with undifferentiated adenocarcinoma. All the patients received postoperative chemotherapy combining cisplatin 60 mg $m^{-2}$, epirubicin 40 mg $m^{-2}$, and cyclophosphamide 300 mg $m^{-2}$ (PAC). In addition, surgical specimens were obtained from 13 women with borderline malignant tumour of the ovary and from 25 women with benign ovarian tumour.

### Immunohistochemical staining of carbonyl reductase

Anti-CR antibody was prepared in a rabbit as reported previously (Kajihara-Kano et al, 1997). All samples surgically obtained for immunohistochemistry were immediately fixed in formaldehyde and embedded in paraffin. Sections 6 μm thick were routinely passed through xylene and a graded alcohol series and stained for CR by the labelled streptavidin biotin method (LSAB kit; DAKO, Santa Barbara, CA) using anti-CR antibody as reported previously (Yokoyama et al, 2000). Anti-CR antibody was applied for 12 h at 4˚C in a moist chamber. The binding sites of peroxidase were determined using 3,3′-diaminobenzidine as the substrate. The sections were then counterstained with haematoxylin for microscopic examination. As negative control, preimmune rabbit serum or antibody preparation absorbed with the antigen was used instead of the antibody. As positive control for CR staining, formalin-fixed paraffin-embedded sections of normal human liver were stained by the same procedure (Suto et al, 1999). 2 observers independently evaluated and interpreted the results of immunohistochemical staining, without knowledge of the clinical data of each patient. Immunohistochemical staining was evaluated according to a scoring method reported previously (Yokoyama et al, 2000). Namely, a score was established corresponding to the sum of: (1) the percentage of positive cells (0, 0% immunopositive cells; 1, < 25% positive cells; 2, 26–50% positive cells; and 3, > 50% positive cells); and (2) the staining intensity (0, negative; 1, weak; 2, moderate; 3, high). The sum for the assigned values of the positive cell percentage (1) and the staining intensity (2) was 6 or less than 6. Scores between 0 and 2 were regarded as negative, scores of 3 and 4 as weakly positive, and scores of 5 and 6 as strongly positive.

### Statistical analysis

The survival curves were calculated by the Kaplan–Meier method, and the statistical significance of differences in the cumulative survival curves between the groups was evaluated by Cox–Mantel test. Other statistical analyses were carried out by Chi-square test or Fisher's exact probability test. A result was deemed significant at $P < 0.05$.

### RESULTS

### Expression of CR in benign tumour, borderline malignant tumour and cancer

The frequencies of expression of CR in benign tumour, borderline malignant tumour and cancer are demonstrated in Table 1. There was no significant difference in the rates of CR positivity between samples of benign tumour and borderline malignant tumour. The incidence of CR expression (weakly positive and strongly positive) in cancers (45 out of 73, 61.6%) was significantly higher than that in benign tumours (8 out of 25, 32.0%) ($P < 0.01$). CR was in general homogeneously stained in the cytoplasm of positive cases (Figure 1).

### Relationship between CR expression and clinico-pathological factors in ovarian cancers

The correlation between CR expression and clinico-pathological factors in ovarian cancers is displayed in Table 2. CR expression had no correlation to clinical stage, histological type and histological grade. On the other hand, there was an inverse correlation between CR expression and RLN metastasis, namely, the CR-positive rate was 35.7% (weakly positive alone) for ovarian cancers with RLN metastasis and 67.8% for those without RLN metastasis, the former being significantly lower than the latter ($P <$ 0.05). Moreover, CR expression in metastatic lymph nodes was weaker with a marginal significance ($P = 0.086$) than that in the primary lesions (Table 3). There was no significant difference in CR expression between the intraperitoneal or pelvic disseminated foci and those primary lesions (Table 3).

Of the 73 patients, 25 had drug resistance, 12 of whom underwent the resection of the remaining or recurrent tumours. There was no significant difference in CR expression between the 12 resected tumours and those primary lesions.

**Table 1** Correlation between expression of carbonyl reductase (CR) and ovarian neoplasms

| Tissues | No. of patients | CR expression (%) | | |
|---|---|---|---|---|
| | | Negative | Weakly positive | Strongly positive |
| Benign tumour | 25 | 17 (68.0) | 7 (28.0) | 1 (4.0) |
| Borderline malignancy | 13 | 8 (61.5) | 3 (23.1) | 2 (15.4) |
| Cancer | 73 | 28 (38.4) | 32 (43.8) | 13 (17.8) |

Statistical significance is described in detail in the text.

### Survival of patients with ovarian cancer according to CR expression

5-year survival rate of patients with CR weakly or strongly positive cancer was 86.1% and that of those with CR-negative one was 62.7%. The prognosis for patients with CR negative cancer was significantly poorer than that for those with CR weakly or strongly positive cancer (Figure 2, $P < 0.05$).

### DISCUSSION

We have recently reported a novel function for CR, namely, its ability to modulate the metastatic potential of malignant mouse cells (Ismail et al, 2000). We have shown that *carbonyl reductase* mRNA was not detectable in the sub-line with high metastatic capacity derived from a mouse lung adenocarcinoma cell line, whereas it was present at high abundance in the sub-line with low metastatic capacity derived from the same one (Ismail et al, 2000). Furthermore, transfection of the low metastatic-potential subline with a construct expressing antisense *carbonyl reductase* rendered the cells highly metastatic, and conversely transfection of the high metastatic-potential subline with a construct expressing sense *carbonyl* reductase decreased their metastatic capacity markedly (Ismail et al, 2000). Recent immunohistochemical examination also demonstrated that decreased expression of CR in hepatocellular cancers was proportional to the degree of dedifferentiation (Suto et al, 1999). In the present study, although intensity of CR expression in epithelial ovarian cancer was unrelated to the histological grade, decrease or loss of CR expression in epithelial ovarian cancer significantly correlated with RLN metastasis. These results suggest an important function for CR in modulating the metastatic potential of malignant tumour.

*© 2001 Cancer Research Campaign*

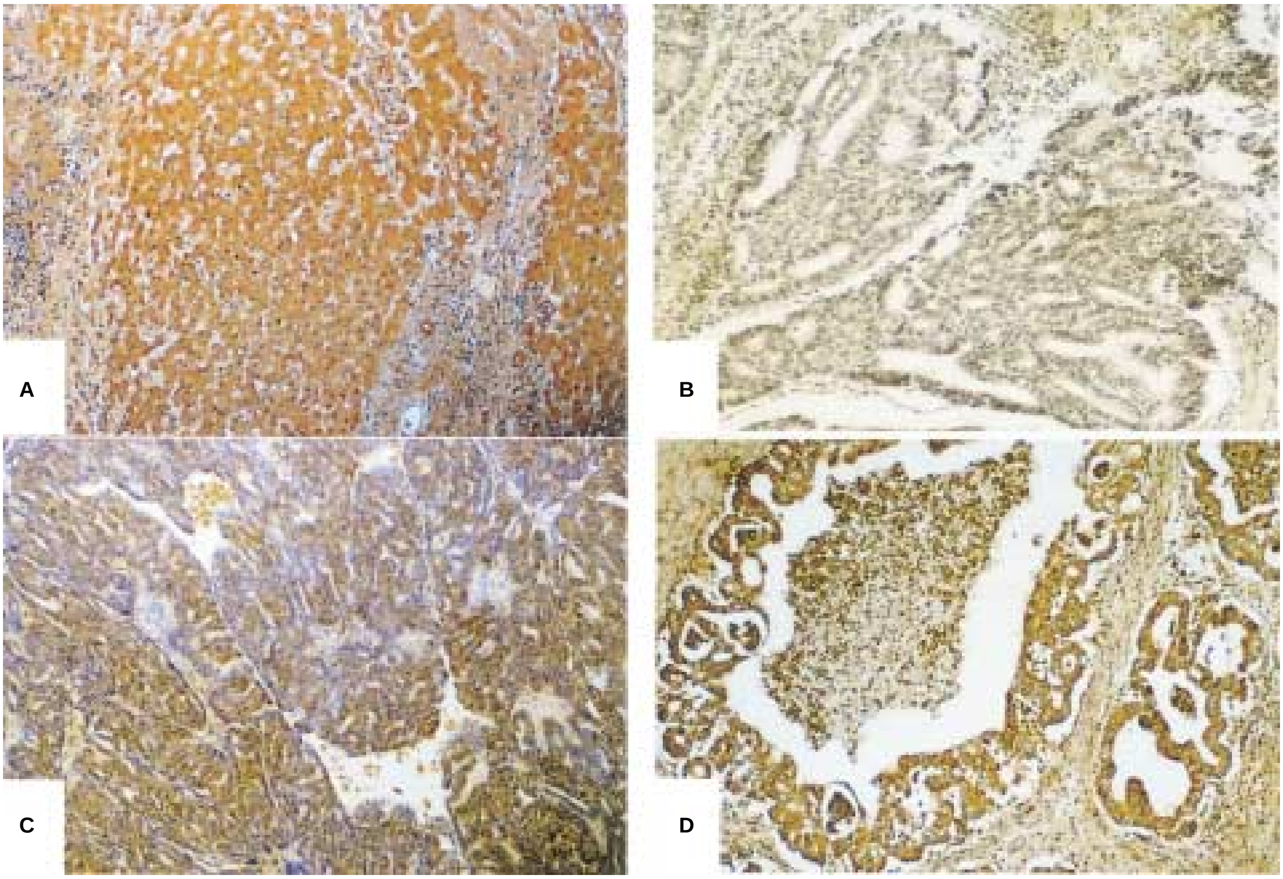

**Figure 1** Immunohistochemical staining of carbonyl reductase in epithelial ovarian cancers. **A**, **B**, **C** and **D** demonstrate positive control in normal liver, negative, weakly positive and strongly positive staining, respectively, in epithelial ovarian cancer tissues. Original magnification, × 200

**Table 2** Relationship between expression of carbonyl reductase and clinico-pathological factors in ovarian cancer

| Factors | No. of patients | CR expression (%) | | |
|---|---|---|---|---|
| | | Negative | Weakly positive | Strongly positive |
| Stage | | | | |
| I and II | 45 | 17 (37.8) | 18 (40.0) | 10 (22.2) |
| III and IV | 28 | 11 (39.8) | 14 (50.0) | 3 (10.7) |
| Histological type | | | | |
| Serous | 30 | 12 (40.0) | 13 (43.3) | 5 (16.7) |
| Mucinous | 17 | 7 (41.2) | 7 (41.2) | 3 (17.6) |
| Endometrioid | 15 | 5 (33.3) | 8 (53.3) | 2 (13.4) |
| Clear cell | 8 | 3 (37.5) | 3 (37.5) | 2 (25.0) |
| Undifferentiated | 3 | 1 (33.3) | 1 (33.3) | 1 (33.3) |
| Grade | | | | |
| Well | 42 | 16 (38.1) | 19 (45.2) | 7 (16.7) |
| Moderate | 16 | 6 (37.5) | 8 (50.0) | 2 (12.5) |
| Poor | 7 | 3 (42.8) | 2 (28.6) | 2 (28.6) |
| Lymph node metastasis | | | | |
| – | 59 | 19 (32.2) | 27 (45.8) | 13 (22.0) |
| + | 14 | 9 (64.3) | 5 (35.7) | 0 |

Statistical significance is described in detail in the text.

CR has been found to be biochemically, immunologically and functionally identical to prostaglandin 9-ketoreductase (PG 9-KR) (Schieber et al, 1992). PG 9-KR oxidises prostaglandin $E_2$, $F_{2\alpha}$ and $D_2$ to biologically inactive 15-keto metabolites (Schieber et al, 1992). Prostaglandins play an important role in modulating tumour growth and metastasis in a variety of human tumours (Ablin and Shaw, 1986). Prostaglandin $E_2$ has been shown to have important functions in inducing angiogenesis (Tsujii et al, 1998) as well as in inhibiting apoptosis (Sheng et al, 1998) in cancer cells. Our recent immunohistochemical study revealed a significant relationship

 *© 2001 Cancer Research Campaign*

**Table 3** Comparison of carbonyl reductase expression between primary lesions and metastatic foci

| | No. of patients | CR expression (%) | | |
|---|---|---|---|---|
| | | Negative | Weakly positive | Strongly positive |
| Primary lesion with intraperitoneal dissemination | 24 | 11 (45.8) | 11 (45.8) | 2 (8.4) |
| Intraperitoneal disseminated foci | 24 | 9 (37.5) | 9 (37.5) | 6 (25.0) |
| Primary lesion with retroperitoneal lymph node metastasis | 14 | 9 (64.3) | 5 (35.7) | 0 |
| Metastatic lymph node | 14 | 12 (85.7) | 2 (14.3) | 0 |

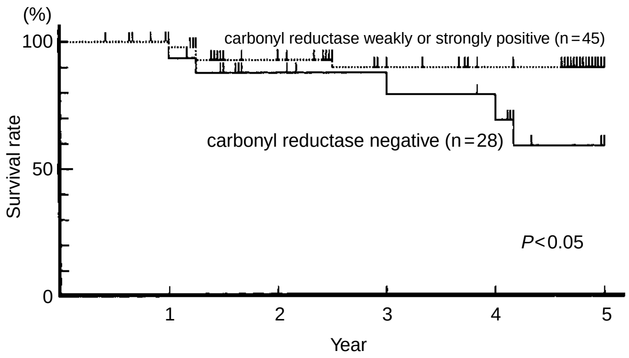

**Figure 2** Survival curves of epithelial ovarian cancer patients according to carbonyl reductase (CR) immunoreactivity. 5-year survival rate of patients with CR weakly or strongly positive cancer was 86.1% and that of those with CR negative one was 62.7%. The latter was significantly lower than the former ($P < 0.05$)

between RLN involvement and the expression of Flt-4 in endometrial cancers and a marginally significant correlation between RLN involvement and VEGF expression, suggesting that VEGF-Flt-4 system might promote lymph-angiogenesis and lymph node metastasis in endometrial cancer (Yokoyama et al, 2000).

CR metabolizes a variety of antitumour drugs containing carbonyl groups, such as the anthracycline antibiotics, daunorubicin and doxorubicin (Ahmed et al, 1978). CR reduces the methyl ketone in the carbon side-chain of daunorubicin to its corresponding alcohol (Kuffel et al, 1992). It has been previously suggested that high CR activity found in some tumour cells might contribute to tumoural resistance to some drugs metabolized in such a way (Lopez de Cerain et al, 1999). The present results, however, demonstrated no significant difference in CR expression between the chemosensitive ovarian cancers and the chemoresistant ones, suggesting that there might be little connection between drug resistance and CR expression in epithelial ovarian cancer. It should be noted that the key anticancer drug for epithelial ovarian cancer treatment is broadly known to be cisplatin, however, it remains to be determined whether CR activity affects metabolism of cisplatin in vivo.

In conclusion, the present results indicate that decreased CR expression in epithelial ovarian cancer tissues is associated with RLN metastasis and poor survival. This is the first study in any human cancer to show a strong correlation between survival and CR level and supports a more important role for CR in human tumours than has been previously thought. Further studies are urgently needed to clarify the precise mechanisms by which CR alters the metastatic potential of cancer cells.

### ACKNOWLEDGEMENTS

This study was supported in part by the Karoji Memorial Fund of the Hirosaki University School of Medicine, by a Grant-in Aid for Cancer Research (No. 12770893) from the Ministry of Education, Science and Culture of Japan, and by a grant from Kuwait Foundation for the Advancement of Sciences number 990707.

### REFERENCES

Ablin RJ and Shaw MW (1986) Prostaglandin modulation of prostate tumor growth and metastases. *Anticancer Res* **6**: 327–328

Ahmed NK, Felsted RL and Bachur NR (1978) Heterogeneity of anthracycline antibiotics carbonyl reductases in mammalian livers. *Biochem Pharmacol* **27**: 2713–2719

Chambers SK, Kacinski BM, Ivins CM and Carcangiu ML (1997) Overexpression of epithelial macrophage colony-stimulating factor (CSF-1) and CSF-1 receptor: a poor prognostic factor in epithelial ovarian cancer, contrasted with a protective effect of stromal CSF-1. *Clin Cancer Res* **6**: 999–1007

Ismail E, Al-Mulla F, Tsuchida S, Suto K, Motley P, Harrison PR and Birnie GD (2000) Carbonyl reductase: a novel metastasis modulating function. *Cancer Res* **60**: 1173–1176

Iwata N, Inazu N and Satoh T (1990) Immunological and enzymological localization of carbonyl reductase in ovary and liver of various species. *J Biochem* **107**: 209–212

Kajihara-Kano H, Hayakari M, Satoh K, Tomioka Y, Mizugaki M and Tsuchida S (1997) Characterization of S-hexylglutathione binding proteins of human hepatocellular carcinoma: separation of enoyl-CoA isomerase from an alpha class glutathione transferase form. *Biochem J* **328**: 473–478

Kuffel MJ, Reid JM and Ames MM (1992) Anthracyclines and their C-13 alcohol metabolites: growth inhibition and DNA damage following incubation with human tumor cells in culture. *Cancer Chemother Pharmacol* **30**: 51–57

Lee SC and Levine L (1974) Prostaglandin metabolism I. Cytoplasmic reduced nicotinamide adenine dinucleotide phosphate-dependent and microsomal reduced nicotinamide adenine dinucleotide-dependent prostaglandin E9-ketoreductase activities in monkey and pigeon tissues. *J Biol Chem* **249**: 1369–1375

Lopez de Cerain A, Marin A, Idoate MA, Tunon MT and Bello J (1999) Carbonyl reductase and NADPH cytochrome P450 reductase activity in human tumoral versus normal tissues. *Eur J Cancer* **35**: 32–34

Schieber A, Frank RW and Ghisla S (1992) Purification and properties of prostaglandin 9-ketoreductase from pig and human kidney: Identity with human carbonyl reductase. *Eur J Biochem* **206**: 491–502

Sheng H, Shao J, Morrow JD, Beauchamp RD and DuBois RN (1998) Modulation of apoptosis and bcl-2 expression by prostaglandin $E_2$ in human colon cancer cells. *Cancer Res* **58**: 362–366

Silverberg SG (1999) Molecular diagnosis and prognosis in gynecologic oncology. *Arch Pathol Lab Med* **123**: 1035–1040

*© 2001 Cancer Research Campaign*

Suto K, Kajihara-Kano H, Yokoyama Y, Hayakari M, Kimura J, Kumano T, Takahata T, Kudo H and Tsuchida S (1999) Descreased expression of the peroxisomal bifunctional enzyme and carbonyl reductase in human hepatocellular carcinomas. *J Cancer Res Clin Oncol* **125**: 83–88

Tempfer C, Obermair A, Hefler L, Haeusler G, Gitsch G and Kainz C (1998) Vascular endothelial growth factor serum concentrations in ovarian cancer. *Obstet Gynecol* **92**: 360–363

Tsujii M, Kawano S, Tsuji S, Sawaoka H, Hori M and DuBois RN (1998) Cyclooxygenase regulates angiogenesis induced by colon cancer cells. *Cell* **93**: 705–716

Wermuth B (1981) Purification and properties of an NADPH-dependent carbonyl reductase from human brain. Relationship to prostaglandin 9-ketoreductase and xenobiotic ketone reductase. *J Biol Chem* **256**: 1206–1213

Wermuth B, Bohren KM, Heinemann G, Von Warthburg JP and Gabbay KH (1988) Human carbonyl reductase. Nucleotide sequence analysis of a cDNA and amino acid sequence of the encoded protein. *J Biol Chem* **263**: 16185–16188

Werness BA, Freedman AN, Piver MS, Romero-Gutierrez M and Petrow E (1999) Prognostic significance of p53 and $p21^{waf1/cip1}$ immunoreactivity in epithelial cancers of the ovary. *Gynecol Oncol* **75**: 413–418

Yokoyama Y, Sakamoto T, Sato S and Saito Y (1999) Evaluation of cytoreductive surgery with pelvic and paraaortic lymphadenectomy and intermittent cisplatin-based combination chemotherapy for improvement of long-term survival in ovarian cancer. *Eur J Gynaecol Oncol* **20**: 361–366

Yokoyama Y, Sato S, Futagami M, Fukushi Y, Sakamoto T, Umemoto M and Saito Y (2000) Prognostic significance of vascular endothelial growth factor and its receptors in endometrial carcinoma. *Gynecol Oncol* **77**: 413–418

*© 2001 Cancer Research Campaign*
